# How to avoid microaspiration? A key element for the prevention of ventilator-associated pneumonia in intubated ICU patients

**DOI:** 10.1186/1471-2334-14-119

**Published:** 2014-11-28

**Authors:** Stijn I Blot, Jan Poelaert, Marin Kollef

**Affiliations:** Dept. of Internal Medicine, Faculty of Medicine & Health Sciences, Ghent University, De Pintelaan 185, 9000 Ghent, Belgium; Burns, Trauma and Critical Care Research Centre, The University of Queensland, Brisbane, Australia; Dept. of Anesthesiology & Perioperative Medicine, University Hospital Brussels, Laarbeeklaan 101, 1090 Brussels, Belgium; Division of Pulmonary and Critical Care Medicine, Washington University School of Medicine, 660 South Euclid Avenue, Campus Box 8052, St Louis, MO 63110 USA

**Keywords:** Ventilator-associated pneumonia, Pneumonia, Micro-aspiration, Prevention, Infection

## Abstract

Microaspiration of subglottic secretions through channels formed by folds in high volume-low pressure poly-vinyl chloride cuffs of endotracheal tubes is considered a significant pathogenic mechanism of ventilator-associated pneumonia (VAP). Therefore a series of prevention measures target the avoidance of microaspiration. However, although some of these can minimize microaspiration, benefits in terms of VAP prevention are not always obvious. Polyurethane-cuffed endotracheal tubes successfully reduce microaspiration but high quality data demonstrating VAP rate reduction are lacking. An analogous conclusion can be made regarding taper-shaped cuffs compared with classic barrel-shaped cuffs. More clinical data regarding these endotracheal tube designs are needed to demonstrate clinical value in addition to in vitro-based evidence. The clinical usefulness of endotracheal tubes developed for subglottic secretions drainage is established in multiple studies and confirmed by meta-analysis. Any change in cuff design will fail to prevent microaspiration if the cuff is insufficiently inflated. At least one well-designed trial demonstrated that continuous cuff pressure monitoring and control decrease the risk of VAP. Gel lubrication of the cuff prior to intubation temporarily hampers microaspiration through sludging the channels formed by folds in high volume-low pressure cuffs. As the beneficial effect of gel lubrication is temporarily, its potential to reduce VAP risk is probably nonsignificant. A minimum positive end-expiratory pressure of at least 5 cmH_2_O can be recommended as it reduces the risk of microaspiration in vitro and in vivo. One randomized controlled study demonstrated a reduced risk of VAP in patients ventilated with PEEP (5–8 cmH_2_O). Regarding head-of-bed elevation, it can be recommended to avoid supine positioning. Whether a 45° head-of-bed elevation is to be preferred above 25-30° head-of-bed elevation remains unproven. Finally, the routine monitoring of gastric residual volumes in mechanically ventilated patients receiving enteral nutrition cannot be recommended.

## Background

Ventilator-associated pneumonia (VAP) remains a feared complication in intensive care unit (ICU) and high-risk surgical postoperative patients [1]. VAP is associated with substantial excess morbidity [2–4] and may suppress survival [5–9]. On average 10-20% of ICU patients ventilated for >2 days experience VAP [10]. However, VAP incidence may vary according to diagnostic approach. Broad-scaled initiatives to streamline definitions and surveillance methods are necessary to allow fair benchmarking [11]. Incidence estimates may also vary with patients’ risk profile and compliance with prevention measures, which remains an issue in many ICUs [12–16].

VAP prevention targets the main pathogenic mechanism, which is bacterial translocation from stomach and oropharynx to the lower respiratory tract. Within hours following endotracheal intubation pathogenic microorganisms colonize the oropharyngeal mucosal surfaces, dental plaque, sinuses, and stomach [17, 18]. Accumulation of oropharyngeal secretions colonized with these pathogens occurs above the endotracheal tube (ETT) cuff. Microaspiration of these subglottic secretions might occur through an underinflated tracheal cuff or through longitudinal folds in high volume-low pressure cuffs. Furthermore, a nasogastric tube may facilitate gastroesophageal reflux. Therefore, gastric juice may be aspirated into the lungs, provoking local inflammation [17, 19]. Generally, the prevention of VAP is focused on reducing the exposure time, maintaining oral hygiene by antiseptic rinsing, and avoidance of microaspiration [20–22]. However, microaspiration seems decisive because it is unlikely that prolonged exposure or bad oral hygiene as such would arouse VAP in the absence of substantial microaspiration. Consequently, a lot of emphasis is given to avoidance or minimization of microaspiration [22]. Several preventive measures have been developed to decrease the risk of microaspiration. Although some of these successfully reduced microaspiration, their potential to reduce VAP is not always proven. The objective of this paper is to briefly review measures to avoid microaspiration of subglottic secretions and to evaluate their effectiveness in doing so and in preventing VAP.

## Review

### Selection of the endotracheal tube

In the 1960s cuffs of ETTs were made of red rubber. While these “high pressure-low volume” cuffs were successful in sealing the extraluminal airway, ischemic damage of the tracheal wall was an issue. Therefore these ETTs were soon replaced by ETTs with “high volume-low pressure” (HVLP) cuffs made out of polyvinyl chloride (PVC). In the past decades HVLP cuff ETTs experienced several changes in design, some of them with the primary aim to reduce the risk of microaspiration. Some of these will be mentioned hereunder.

#### Endotracheal tube cuff material: polyurethane vs. PVC

One of the disadvantages of the standard HVLP cuff is that the diameter of the cuff is bigger than the tracheal diameter. As such, only a low pressure is needed for inflation and for letting the cuff adapt to the shape of the trachea. With this concept, however, the cuff is not fully unfolded resulting in channel formation along the cuff. Through these channels microaspiration might occur.

Ultrathin polyurethane cuffs have been developed to minimize the channel size within folds of an inflated cuff. In an in vitro setup using a tracheal model with a 20 mm internal diameter, fluid leakage past the tube cuff was compared between a polyurethane cuff (cuff membrane thickness 7 μm) and four ETTs with a PVC cuff (cuff membrane thickness 50 to 70 μm) [23]. Fluid leakage was evaluated at cuff pressures of 10, 15, 20, 25, 30 and 60 cmH_2_O, and the amount of fluid leakage was recorded at 5, 10, and 60 minutes. Within the recommended target cuff pressure of 20–30 cmH_2_O, the polyurethane cuff was the only to effectively prevent fluid leakage past the cuff. In another in vitro study three types of polyurethane cuffed ETTs were compared with three types of ETTs with a PVC cuff [24]. The amount of fluid leakage after 1 hr was evaluated in three artificial tracheal models varying in internal diameter (16, 20, and 22 mm). Overall, polyurethane ETTs prevented fluid leakage more efficiently than PVC cuffs (p < 0.001). Similar observations were made in a bench-top study in which tracheal models were exposed to different levels of positive end-expiratory pressure (PEEP) [25].

Concerning clinical outcomes, the available data are scarce. A polyurethane cuffed ETT significantly reduced the risk of early post-operative pneumonia in high-risk cardiac surgical patients from 42% to 23% (adjusted odds ratio 0.31, 95% confidence interval [CI] 0.13–0.77) [26]. In a randomized controlled trial, Mahmoodpoor et al. compared rates of VAP associated with three types of ETTs: a cylindrical polyurethane cuffed, a taper-shaped polyurethane cuffed, and a cylindrical PVC cuffed tube [27]. In a comparison with polyurethane cuffed tubes (both groups merged) and the PVC cuffed ETT, no difference in VAP could be observed (20% vs. 34%, respectively; p = 0.134). However, this study suffers several limitations. The study was likely to be underpowered and VAP was defined solely in accordance with the clinical pulmonary infection score thereby probably leading to overestimation of the true incidence.

#### Endotracheal cuff shape: tapered vs. cylindrical

Conventional ETTs have a cylindrical-shaped cuff. An ETT with a taper-shaped cuff was developed with the promise to better adapt to natural variations in the size of the trachea. Because of its tapered shape, this cuffs seals the trachea, at least at one point, without fold formation. An in-vitro study was able to demonstrate superior sealing capacity of these taper-shaped cuffs compared to cylindrical-shaped cuffs [24]. Additionally, they appeared to be equally effective in preventing fluid leakage as cylindrical-shaped polyurethane cuffed ETTs. Of note, in tracheal models with a larger diameter the favorable effect of taper-shaped cuffs over cylindrical-shaped polyurethane cuffs was greater. In a clinical bronchoscopy-controlled study in patients undergoing lumbar surgery, microaspiration of instilled methylthionium chloride was compared between taper-shaped PVC cuffs and barrel-shaped PVC cuffs [28]. After 30 minutes, following turning the patients in prone position, the barrel-shaped cuff showed descent of dye into the trachea in 20% of the patients. Contrariwise, after two hours of observation, no dye leakage into the trachea was observed with taper-shaped cuffs. To what extent the endotracheal tube with a taper-shaped cuff results in reduced VAP rates remains to be demonstrated.

#### Subglottic secretions drainage (SSD)

Another strategy to prevent microaspiration is to avoid accumulation of subglottic secretions above the cuff. ETTs for SSD can drain secretions through a separate dorsal lumen that opens directly above the cuff. A meta-analysis pooling 13 randomized controlled trials and encompassing 2442 patients demonstrated an overall risk reduction with use of SSD of 0.55 (95% CI, 0.46-0.66) [29]. When only high quality trials were taken into account the effect remained statistically significant (risk ratio 0.54, 95% CI, 0.40-0.73). Overall, the use of SSD was associated with a reduced ICU stay, decreased length of ventilatory dependence, and an increased time to first episode of VAP. Drainage can be successfully performed either continuously or intermittently [30].

### Gel lubrication of the cuff

Gel lubrication of the cuff prior to intubation is mainly done to smoothen the procedure. Yet, by doing so the channels along the cuff wall are plugged thereby blocking microaspiration of oropharyngeal secretions. Blunt et al. compared fluid leakage in lubricated and nonlubricated cuffs in a benchtop model with use of a static pig trachea model [31]. After 15 min. all five nonlubricated cuffs leaked, while in none of the lubricated cuffs dye leakage was observed. Another in vitro study testing six different brands of ETTs revealed that in case of gel lubrication no dye leakage occurred in the 1 hr observation period [32]. In all six nonlubricated cuffs leakage became obvious within five minutes.

Microaspiration in lubricated vs. nonlubricated cuffs was evaluated in a double-blinded, randomized clinical study involving anesthetized patients undergoing extraction of wisdom teeth [31]. In all patients diluted blue food dye was instilled above the cuff after intubation. During the surgical procedure cuff pressure was maintained at 30cmH_2_O by a constant cuff pressure inflator. Microaspiration was evaluated by means of endotracheal aspiration after surgery and before extubation. Microaspiration was witnessed in 11% of lubricated cuffs vs. 83% of nonlubricated cuffs (p < 0.001). A similar approach of blue dye instillation was used in a prospective observational study involving tracheotomized patients with lubricated cuffs only [31]. The lubricated cuffs leaked after a median period of 48 hrs (ranging 24 to 120 hrs). From these studies it appears that microaspiration can be temporarily avoided by gel lubrication. Therefore, gel lubrication might be of value in short term-ventilated patients. However, any value in terms of pneumonia prevention remains unproven.

### Cuff pressure monitoring

No cuff seals when insufficiently inflated. The recommended cuff pressure for HVLP cuffs ranges 20–30 cmH_2_O. However, cuff pressure easily deviates outside this target interval due to pathophysiological and environmental circumstances, and body position changes [33, 34]. Devices to provide automated monitoring and adjustments of cuff pressure have been developed. In an initial randomized controlled trial these devices proved successful to maintain cuff pressure within the target limits, but no effect on VAP rate was observed [35]. It has been suggested that no difference in VAP rate was observed as randomization took place two days following intubation. In another randomized control trial, Nseir et al. also demonstrated that continuous cuff pressure monitoring was effective in maintaining the pressure within the target limits compared with manual control per 8 hours: 98% of measurements *vs*. 74% (p < 0.001) [36]. In addition, patients in the intervention group had a decreased risk for micro-aspiration of gastric contents and VAP (9.8% *vs*. 26.2%; p = 0.032), thereby confining the controversial status of continuous cuff pressure monitoring as valuable to prevent pneumonia [37].

### Positive End-Expiratory Pressure (PEEP)

Experimental studies with HVLP cuffs showed that, as airway pressures rise, the gas contained within the cuff is redistributed from the distal to the proximal cuff end. This results in a cone-shaped cuff in which the intra-cuff pressure is temporarily (during the inspiratory phase) higher than the cuff pressure during the expiratory phase. As such, positive pressure ventilation creates a ‘self-sealing’ effect by which tracheal occlusion is maintained despite airway pressure exceeding intra-cuff pressures [38]. Therefore it was hypothesized that PEEP could result in a better sealing capacity throughout the ventilation cycle, and as such reduce micro-aspiration. In a benchtop study Ouanes et al. demonstrated that microaspiration occurring within one hour decreased from 91% with zero PEEP to 8% with 15 cmH_2_O PEEP [39]. Similarly, also Pitts et al. observed that micro-aspiration decreased with higher levels of PEEP (5, 10 or 15 cmH_2_O) [40]. In this study peak inspiratory pressure also was inversely associated with leakage volume but not anymore when PEEP was set at 15 cmH_2_O. In vitro data by Zanella also indicated that micro-aspiration did not occur within 24 hrs when PEEP was set at 15 cmH2O, irrespective of which ETT type was investigated [25].

Lucangelo et al. evaluated the leakage of dye past the cuff in a bronchoscopy-controlled study in ventilated ICU patients [41]. The experiment lasted for 12 hrs. In the first 5 hrs PEEP was maintained at 5 cmH2O; thereafter PEEP was removed. In two on 40 patients leakage of dye occurred before removal of PEEP (5.0%). After PEEP was removed, leakage became obvious in 37/40 patients (92.5%). A single center trial, non-hypoxic mechanically ventilated patients were randomized to receive either 5–8 cmH_2_0 PEEP (n = 66) or no PEEP (n = 65) [42]. VAP rate among patients ventilated with PEEP was 9.4% and significantly lower compared with the control group (25.4%)(relative risk [RR] 0.37; 95% CI 0.15-0.84). Of note, patients in the intervention group experienced less hypoxemia and there was no difference between the groups in rates of acute respiratory distress syndrome, barotrauma, or atelectasis. In the absence of particular contraindications, the use of at least 5 cmH_2_O of PEEP can be recommended as standard in mechanically ventilated patients. In patients with overt right ventricular failure however, safety of external PEEP could be questioned. In these cases risks and benefits should be carefully considered.

### Semirecumbent position

In 1999 Draculovic reported a lower risk of clinically suspected VAP (RR 0.23, 95% CI 0.07-0.72) and microbiologically documented VAP (RR 0.22, 95% CI 0.05-0.93) among patients cared for in semirecumbent positioning (45° head-of-bed elevation) compared to patients kept in supine position (0° head-of-bed elevation)[43]. Especially patients in the supine group receiving enteral nutrition experienced a high risk of VAP. Another randomized study comparing 45° *vs*. 25° head-of-bed elevation found a nonsignificant reduction (RR 0.38, 95% CI 0.04-3.77) but the study was toughly underpowered (17 vs. 13 patients respectively) [44]. Both studies suffered several limitations such as prematurely stopping after interim analysis [43], high dropout rates [44], and uncertainties about diagnostic approaches [43, 44]. In the Draculovic trial, correctness of patients’ position was checked once daily, while Keeley et al. did not report posture checks. This is a serious flaw. van Niewenhoven et al. conducted a trial in which patients were randomly allocated to a 45° vs. 10° head-of-bed elevation [45]. Head-of-bed elevation was continuously monitored by means of a transducer with pendulum and a dedicated nurse controlled patients’ position twice-to-thrice daily and restored to the target position (if possible). As 85% of the time semirecumbent (45°) positioning as not achieved, the study turned out to be a comparison between approximately 10° *vs*. 28° head-of-bed elevation. No difference in VAP risk was observed.

Although based on the results of a limited-quality trial, most recommendations agree that supine positioning is to be avoided [46]. However, it remains unproven whether 45° head-of-bed elevation is superior to 25-30° elevation. Despite the absence of a clear advantage, an expert panel recommended semirecumbent positioning weighting its potential benefits and harms [47]. Patients at risk for hemodynamic instability following 45° head-of-bed elevation may benefit from a 20-30° backrest elevation [48].

### Monitoring gastric overdistention

Gastric overdistention has been historically considered a risk factor for VAP as it is assumed to facilitate bacterial translocation from the stomach to the respiratory tract. With enteral nutrition becoming standard of care in mechanically ventilated patients monitoring gastrointolerance to enteral feeding by checking residual volumes is frequent practice. Most frequent thresholds used to interrupt enteral feeding are residual volumes of 200–250 mL [49]. Yet, cessation of enteral feeding is not recommended unless residual volumes exceed 500 mL [50]. In addition, monitoring residual gastric volumes may increase the risk of inadequate caloric intake. The effect of not monitoring residual gastric volumes on risk of VAP was evaluated in a multicentre, randomized controlled trial [51]. VAP rate in the absence of monitoring residual volumes was 16.7% and 15.8% in the control group in which residual volumes were checked every 6 hrs (and in which residual volumes greater than 250 mL were returned to the patient) (difference, 0.9%, 90% CI, -4.8-6.7%). No differences were observed between the groups regarding rates of other healthcare-associated infections, length of ventilator dependence, ICU stay, or mortality. Importantly, the proportion of patients receiving 100% of their caloric intake target was significantly higher in the intervention group (odds ratio 1.77, 95% CI, 1.25-2.51). As such, monitoring gastric overdistention seems not to benefit prevention of VAP.

### Interventions indirectly affecting the risk associated with microaspiration

Some interventions do not directly target microaspiration. Their practice however, may alter the risk of microaspiration and/or pneumonia. Based on the available evidence their use can be either be advocated or not. Small bowel feedings, for example, are assumed to minimize the risk of aspiration should intolerance to enteral feedings occur. No data however to support this practice with the aim to reduce VAP risk are available. Routine changes of ventilator circuits do not decrease VAP risk. Even stronger, where no benefit is to be expected the patient may experience a microaspiration by unnecessary manipulation of the tubings.

Mouthwashes with chlorhexidine solutions and selective oral decontamination do not as such reduce the risk of microaspiration [21, 52]. Yet, these interventions reduce the microbial burden in the oral cavity and therefore the inoculum of potential pathogenic microorganisms entering the lower respiratory tract in case of microaspiration.

## Conclusions

Several interventions have been developed in order to reduce the risk of microaspiration and subsequently VAP. Taking into account the effectiveness of avoiding microaspiration and VAP the following measures should be considered: (i) the use of ETT designed for SSD, (ii) continuous cuff pressure monitoring and control, (iii) a minimum PEEP of 5 cmH_2_O, and (iv) avoidance of supine positioning. The following measures lack data to demonstrate their benefits in terms of VAP risk reduction, but are nevertheless interesting because of their potential to reduce microaspiration: (i) gel lubrication of the cuff prior to intubation, (ii) polyurethane cuffed ETTs, and (iii) taper-shaped ETT cuffs.

## Authors’ information

SB is Professor at the Dept. of Internal Medicine at Ghent University and holds a research mandate of the Special Research Fund at Ghent University. JP is Professor and head of the Dept. of Anesthesiology & Peri-Operative Medicine at University Hospital Brussels. MK is Golman Professor of Pulmonary and Critical Care Medicine at Washington University School of Medicine.

## Abbreviations

CI: Confidence interval
ETT: Endotracheal tube
ICU: Intensive care unit
PEEP: Positive end-expiratory pressure
PVC: Polyvinyl chloride
RR: Relative risk
SSD: Subglottic secretions drainage
VAP: Ventilator-associated pneumonia.
